# Assessment of Perioperative Sleep Characteristics Using Subjective and Objective Methods: A Secondary Analysis of Prospective Cohort Study

**DOI:** 10.1155/2023/9633764

**Published:** 2023-04-21

**Authors:** Junyong In, Eunjung Lim, Sakura Kinjo

**Affiliations:** ^1^Department of Anesthesiology and Pain Medicine, Dongguk University Ilsan Hospital, Goyang, Republic of Korea; ^2^Department of Quantitative Health Sciences, John A. Burns School of Medicine, University of Hawaii, Honolulu, Hawaii, USA; ^3^Department of Anesthesia and Perioperative Care, University of California San Francisco, San Francisco, California, USA

## Abstract

Perioperative sleep disturbances may impact healing and negatively affect the patient's perception of well-being. Therefore, accurately assessing postoperative sleep characteristics is necessary to treat sleep disturbances. This study is a secondary data analysis of research investigating the association between sleep and cognition in a perioperative setting. This study compares sleep characteristics between the St. Mary's Hospital Sleep Questionnaire and WatchPAT, a portable sleep apnea testing device. The goal of this study is to compare an objective measurement of sleep quality (WatchPAT) with a traditional questionnaire. One hundred and one patients who underwent elective, noncardiac surgical procedures wore a WatchPAT and completed the St. Mary's Hospital Sleep Questionnaire for three nights: two preoperative and one postoperative night. In the preoperative period, a Bland-Altman analysis showed an agreement Watch PAT and the St Mary's hospital sleep questionnaire except for sleep fragmentation. A good to fair correlation during the preoperative period was observed with both sleep latency and total sleep time. In the postoperative period, no correlation was observed between the St. Mary's Hospital Sleep Questionnaire data and WatchPAT data. Our study indicates that some potential factors affecting sleep and cognition such as admission type, depression, anesthesia type, and sleep apnea may limit patients' ability to report their sleep characteristics after surgery. Therefore, relying solely on one sleep assessment method is not advisable.

## 1. Introduction

Sleep disruption after surgery is a common phenomenon, especially after major surgery [1, 2]. It is characterized by longer sleep latency, increased awakenings, lower total sleep time, loss of rapid eye movement (REM) sleep, disruption of circadian rhythm, and other issues [1–3]. In addition, sleep is an essential physiological function that allows our bodies to rest and restore; hence, such disruptions may affect the body's ability to heal and negatively impact patients' perception of well-being.

Given the number of patients who undergo surgical procedures at some point in their lives, accurately assessing sleep characteristics in the perioperative setting is important. Patient-reported, subjective sleep measures often play an important role in this assessment due to their ease of use. They are efficient, cost-effective, and noninvasive. Plus, they are well-suited to repeated use, which can capture sleep changes for a patient over time [4].

St. Mary's Hospital Sleep Questionnaire (SMHSQ), one of the most commonly used sleep surveys, was developed as a standardized evaluation of sleep experience in the hospital setting, evaluating sleep characteristics during a previous 24-hour period [4] (see the Appendix). This quick 14-question test is simple to administer and designed to be used throughout a hospital stay. It asks patients to describe their sleep over the past 24 hours using subjective rankings. When administered over multiple days, it has been shown to provide a picture of sleep changes in inpatients [4]. However, to the best of our knowledge, the SMHSQ has yet to be well studied by comparing it to objective methods in a perioperative setting. Thus, this study was aimed at evaluating the correlation between SMHSQ as a subjective method and WatchPAT (Itamar Medical, Caesarea, Israel), as an objective method, along with the covariates which may affect sleep characteristics in a perioperative setting.

## 2. Materials and Methods

The present study is a secondary data analysis of a prospective study investigating the association between sleep and cognition in a perioperative setting. The prospective study was conducted from 2013 to 2018 at the University of California, San Francisco Medical Center. The Institutional IRB approved the study, and written informed consent was obtained preoperatively from each patient.

### 2.1. Participants

**Figure 1:**
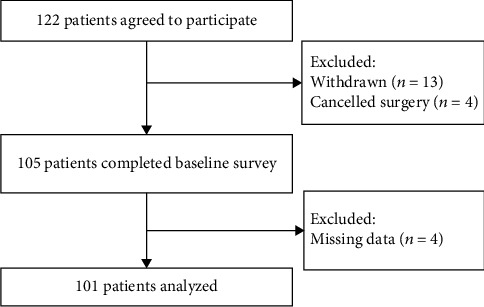
Study flow diagram.

Patients ≥ 50 years of age with a scheduled elective noncardiac procedure were screened for eligibility (Figure 1). Study exclusion criteria included pregnancy, dementia, tumor, peripheral vascular disease, peripheral neuropathy, cardiac disease, pulmonary disease, severe diabetes, auditory or visual handicaps, and surgeries involving the airways, fingers, or arms. In addition, permanent pacemakers, nonsinus cardiac arrhythmias, and short-acting nitrates or alpha-blockers were excluded due to possible interaction with the portable sleep monitoring device, WatchPAT.

The preoperative interview was conducted by a trained research assistant in the preoperative anesthesia clinic, typically less than two weeks and a minimum of 3 days before the patient's surgery, to ensure adequate time for preoperative baseline data collection. We obtained the patient's health information and any potential covariates associated with sleep and cognition, including age, gender, body mass index, race, level of education, American Society of Anesthesiologists status, and preoperative depression-associated symptoms with the Center for Epidemiologic Studies Depression (CES-D) scale [5]. Individuals with a CES-D scale of 16 or greater appear to be at risk for depression.

### 2.2. Sleep Assessments

#### 2.2.1. Objective Sleep Assessment (WatchPAT)

WatchPAT is an FDA-approved portable sleep apnea testing device. It records the parameters of sleep architecture and sleep-disordered breathing using built-in actigraphy and peripheral arterial tonometry. Among the recorded parameters, sleep fragmentation, sleep latency, and total night sleep time were obtained for analysis.

WatchPAT provides an accurate and clinically effective monitoring method; there are high correlations of pAHI, lowest oxygen saturation, and sleep time between polysomnography and the WatchPAT [6, 7].

After obtaining written consent for the study, patients were provided a WatchPAT. Participants wore a WatchPAT on their nondominant hand for two consecutive nights before surgery. On the day of their surgical procedure, patients returned the WatchPAT for analysis. The patients were given a new WatchPAT and asked to wear it on the night of surgery. After surgery, a WatchPAT was used either in the hospital room or at their own homes if they were discharged on the same day of surgery.

#### 2.2.2. Subjective Sleep Assessment (SMHSQ)

Each morning, patients were asked to complete an SMHSQ about their previous night's sleep. Sleep architecture was compared between WatchPAT and SMHSQ using three factors (sleep fragmentation, sleep latency, and total night sleep time). (1) Fragmentation: number of wakes vs. How many times did you wake up? (2) Sleep latency (in minutes) vs. How long did it take you to fall asleep last night. (3) Total night sleep time vs. How much sleep did you have last night (in hours)?

### 2.3. Intraoperative Data

Surgery duration and anesthesia type were noted. Anesthesia types were general, spinal, epidural, and/or peripheral nerve blocks, and then, they were dichotomized as general vs. other.

### 2.4. Postoperative Assessment

For inpatients, postoperative interviews were conducted in the patient's hospital room by the same research assistant for the first postoperative day. For outpatients, patients were contacted by phone following surgery.

### 2.5. Statistical Analysis

Patients' sleep characteristics were summarized using frequency, percentages, means, and standard deviations. Sleep assessment data measured for the two preoperative nights were averaged. If the sleep fragmentation occurred more than 6 times per night with WatchPAT, we used 7 as the top value for analysis in order to correlate with SMHSQ. Pearson's correlation coefficient (*r*) and partial correlation coefficient (partial *r*) controlling for admission type, depression, anesthesia type, and surgery duration were calculated to assess bivariate associations of sleep fragmentation, sleep latency, and total night sleep time between SMHSQ and WatchPAT. Paired *t* tests were conducted to see if there are any differences in the mean values of sleep fragmentation, sleep latency, and total night sleep time between SMHSQ and WatchPAT. In addition, Bland-Altman plots were created to evaluate the agreement of patients' sleep characteristics between the SMHSQ and WatchPAT.

For each sleep assessment, their sleep characteristic data, pre- vs. postsurgery, was analyzed using a mixed-effects model [8, 9]. This model used the following factors: admission type [10, 11], anesthesia type [1, 2], depression [12], obesity [13, 14], and pAHI [15]. In this model, the patient was treated as a random effect. All analyses were performed using R version 4.1.2 (R Foundation for Statistical Computing, Vienna, Austria), and a two-sided *p* value of <0.05 was considered significant.

## 3. Results

### 3.1. Descriptive Data

A total of 101 patients were included in this study, with a mean age of 58.1 ± 11.3 years.  Table 1 shows patients' baseline characteristics. Sixty patients (59.4%) were male. Most patients were admitted after surgery (71.3%), and 59.4% received general anesthesia.

### 3.2. Correlation and Agreement between WatchPAT and SMHSQ

**Figure 2:**
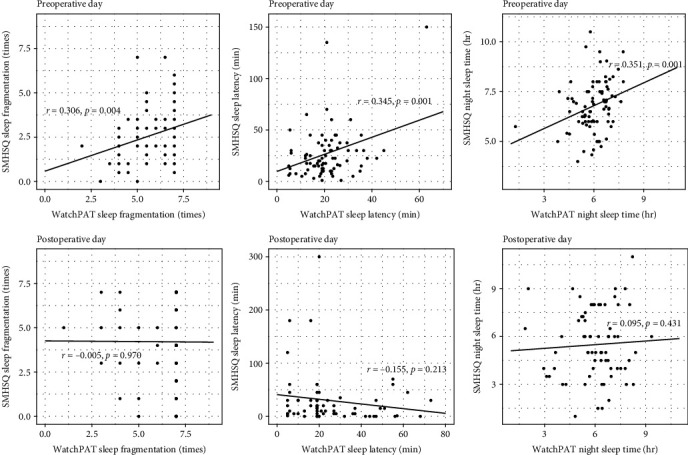
Scatter plots of sleep fragmentation, sleep latency, and total night sleep before and after surgery. *r*: Pearson's correlation; SMHSQ: St. Mary's Hospital Sleep Questionnaire.

**Figure 3:**
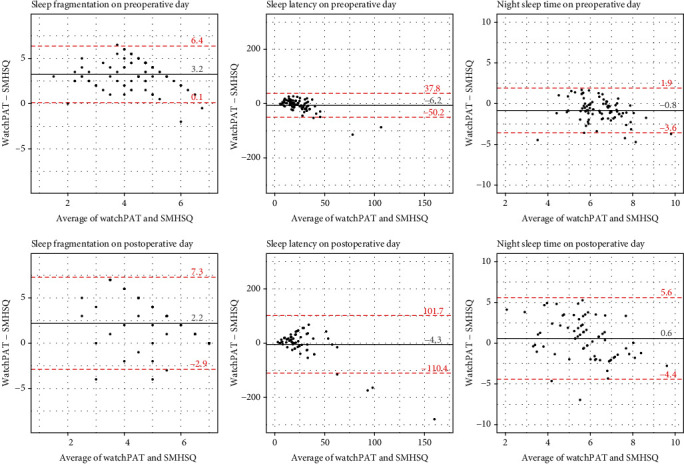
Bland-Altman plots of sleep fragmentation, sleep latency, and total night sleep before and after surgery. SMHSQ: St. Mary's Hospital Sleep Questionnaire. In each plot, the solid black line represents the mean difference (bias), and the red dashed lines represent the 95% limits of agreement of the mean difference.

Positive correlations were found in terms of sleep fragmentation (*r* = 0.31, partial *r* = 0.34), sleep latency (*r* = 0.34, partial *r* = 0.33), and night sleep time (*r* = 0.35, partial *r* = 0.33) between the SMHSQ and WatchPAT data before surgery. However, there were no significant correlations between WatchPAT and the SMHSQ data for sleep fragmentation (*r* = 0.00, partial *r* = −0.10), sleep latency (*r* = −0.16, partial *r* = −0.03), and total night sleep time (*r* = 0.09, partial *r* = −0.01) on the night of surgery (Table 2 and Figure 2). The mean values in each variable were compared using *t* tests. They were statistically different in all three areas before surgery. After surgery, the mean values were similar except for sleep fragmentation (Table 2).  Figure 3 depicts Bland-Altman plots for these sleep characteristics. For the preoperative period, all points were randomly spread around the mean difference, and almost all points were within generally accepted limits of agreement. The mean differences in sleep latency and total night sleep were close to zero, indicating a good agreement between WatchPAT and SMHSQ. However, the average difference in sleep fragmentation between the WatchPAT value was 3.2 times different than the SMHSQ value, indicating that the two methods were significantly different from zero. No significant correlations were noted in all parameters in the postoperative period.

### 3.3. Effects of Various Covariates on Each Measurement Method

Table 3 presents the results of the mixed-effects models controlling for pre-/postoperative night, admission type (inpatient vs. outpatient), anesthesia (general vs. other), depression (CES − D ≥ 16 vs. <16), obesity (BMI ≥ 30 vs. <30), and sleep apnea (pAHI ≥ 15 vs. <15).

#### 3.3.1. Postoperative Night vs. Preoperative Night

1. Sleep fragmentation: WatchPAT showed an increase in sleep fragmentation of 0.50 episodes per night (95% CI = [0.13, 0.87], *p* = 0.007) postoperatively, compared with preoperative sleep fragmentation. The SMHSQ also reported an increased sleep fragmentation, but significantly higher, at 1.50 episodes (95% CI = [0.96, 2.04], *p* < 0.001) postoperatively
2. Sleep latency: WatchPAT showed an increase of 3.85 minutes (95% CI = [0.17, 7.56], *p* = 0.040) postoperatively. The SMHSQ did not show any differences between pre- and postoperative nights (95% CI = [−10.16, 12.78], *p* = 0.8)
3. Total night sleep time: WatchPAT did not show any differences between pre- and postoperative nights (95% CI = [−0.30, −0.37], *p* = 0.8), but the SMHSQ data showed a significant decrease in total night sleep time of 1.51 hours (95% CI = [−2.07, −0.94], *p* < 0.001) after surgery, as reported by the patients

#### 3.3.2. Inpatient vs. Outpatient

1. Sleep fragmentation: WatchPAT showed a decreased sleep fragmentation of 0.79 episodes per night (95% CI = [−1.34, -0.24], *p* = 0.005) for inpatients vs. outpatients. However, no significant difference was noted on the SMHSQ (95% CI = [0.96, 2.04], *p* = 0.15)
2. Sleep latency: no difference was noted in sleep latency on the WatchPAT (95% CI = [−4.74, 6.89], *p* = 0.7) and SMHSQ (95% CI = [−12.94, 17.22], *p* = 0.8) for inpatients vs. outpatients
3. Total night sleep time: for inpatients vs. outpatients, total sleep time increased by 0.75 hours (95% CI = [0.26, 1.25], *p* = 0.003) based on the WatchPAT data. This difference, however, was not apparent based on the SMHSQ data (95% CI = [−1.31, 0.19], *p* = 0.15)

#### 3.3.3. General Anesthesia vs. Other

1. Sleep fragmentation: patients who received general anesthesia had less sleep fragmentation of 0.60 episodes/night (95% CI = [−1.12, −0.08], *p* = 0.025) after surgery than patients who received other types of anesthesia based on WatchPAT data, but no difference was noted on the SMHSQ (95%CI = [−0.60, 0.88], *p* = 0.7)
2. Sleep latency: sleep latency was not significantly different after surgery, comparing general anesthesia and other types of anesthesia on WatchPAT (95% CI = [−4.97, 6.01], *p* = 0.9) and SMHSQ data (95% CI = [−21.04, 7.05], *p* = 0.3)
3. Total night sleep time: on WatchPAT, sleep time was longer by 0.56 hours (95% CI = [0.09, 1.03], *p* = 0.019) in patients who received general anesthesia than in patients who had other types of anesthesia. This difference was not apparent in the SMHSQ data (95% CI = [−1.31, 0.9], *p* = 0.2)

#### 3.3.4. Depression (CES − D ≥ 16 vs. CES − D < 16)

1. Sleep fragmentation: a decrease in sleep fragmentation of 0.91 episodes per night (95% CI = [−1.67, −0.14], *p* = 0.020) was detected in the patients with depression on the SMHSQ but not apparent in the WatchPAT data (95% CI = [−0.64, −0.45], *p* = 0.7)
2. Sleep latency: no significant changes were noted with either reporting method (WatchPAT: 95% CI = [−0.30, 11.13], *p* = 0.063, SMHSQ: 95% CI = [−2.46, 26.86], *p* = 0.10)
3. Total night sleep time: no significant changes were noted in the WatchPAT (95% CI = [−0.70, 0.27], *p* = 0.4) and SMHSQ data (95% CI = [−1.14, 0.32], *p* = 0.3)

#### 3.3.5. Obesity (BMI ≥ 30 vs. <30)

1. Sleep fragmentation: no significant changes were noted in either dataset (WatchPAT: 95% CI = [−0.42, 0.64], *p* = 0.7, SMHSQ: 95% CI = [−0.39, 1.12], *p* = 0.3)
2. Sleep latency: in the BMI ≥ 30 group, sleep latency decreased by 6.08 minutes (95% CI = [−11.64, −0.52], *p* = 0.032) based on WatchPAT data. Reported sleep latency showed an increase of 15.15 minutes (95% CI = [0.63, 29.67], *p* = 0.041) based on the SMHSQ data
3. Total night sleep time: it was decreased for 0.54 hours (95% CI = [−1.01, −0.06], *p* = 0.026) in the BMI ≥ 30 group, but SMHSQ did not report any significant differences in minutes (95% CI = [−1.40, 0.02], *p* = 0.057)

#### 3.3.6. Sleep Apnea (pAHI ≥ 15 vs. <15)

1. Sleep fragmentation: sleep apnea was not associated with changes in sleep fragmentation in either the WatchPAT (95% CI = [−0.18, 0.83], *p* = 0.2) or SMHSQ data (95% CI = [−0.60, 0.83], *p* = 0.8)
2. Sleep latency: WatchPAT (95% CI = [−1.23, 9.33], *p* = 0.13) and the SMHSQ data (95% CI = [−20.97, 6.38], *p* = 0.3) did not show any clear differences
3. Total night sleep time: data for total night sleep time showed an increase of 0.70 hours (95% CI = [0.02, 1.37], *p* = 0.042) in the pAHI ≥ 15 group, using the SMHSQ assessment. This increase was not apparent in the WatchPAT data (95% CI = [−0.16, 0.74], *p* = 0.2)

## 4. Discussion

In this cohort study, comparing both subjective and objective methods, good to fair correlations were observed before surgery for sleep fragmentation, sleep latency, and total night sleep time. However, there were no meaningful correlations after surgery. Similarly, the Bland-Altman analysis showed no correlation between the two methods in the postoperative period.

Literature suggests that multiple factors can affect sleep quality after surgery: environment, physical/pathophysiological, and psychological [2, 16, 17]. The main environmental factors are the unfamiliar environment, nurses' footsteps, and voices of other patients. Physical/pathophysiological factors include dyspnea, pain, and foreign body sensation caused by intubation. An example of a psychological factor is anxiety about unpredictable visits and examinations by a doctor or nurse [16].

### 4.1. Comparison to Previous Studies

Previous studies have investigated sleep using the SMHSQ in non-perioperative settings [18–20], and the SMHSQ was found to be a valuable tool. For example, Jared et al. compared the SMHSQ and the scaled General Health and Hillier Questionnaire in patients with cystic fibrosis. They concluded that these two subjective methods were well correlated. However, the SMHSQ has yet to be well studied in the perioperative setting. Our findings indicate that the WatchPAT and SMHSQ do not correlate well after surgery. Comparing the mean values, WatchPAT showed more sleep fragmentation than the SMHSQ data postoperatively. It is possible that patients could recall full awakenings only, not shorter or less powerful arousals.

We speculate that changes in sleep patterns and cognitive dysfunction after surgery may contribute to discrepancies between the two methods. Therefore, we further explored the factors which may affect sleep and cognition: pre-/postoperative night, admission type, depression, anesthesia type, and sleep apnea [2, 21, 22] using the mixed-effects model (Table 3). Our study indicates that all these factors showed a discrepancy in at least one of the three variables. To the best of our knowledge, the previous studies have yet to investigate the etiology of a discrepancy between the SMHSQ and objective methods.

There are some limitations to our study. First, our sleep data was limited to night sleep only and did not include sleep happening during the day. The study was conducted this way since the WatchPAT is meant to be worn at night. Second, the sample size of our study was relatively small, and there needed to be more data to evaluate more than one night after surgery. To evaluate covariates and other unknown variables affecting perioperative sleep, studies with an appropriate sample size should be conducted in the future. Third, this study did not use polysomnography due to its resource intensiveness in the perioperative period. Although WatchPAT is significantly easier to use for sleep evaluation and has a high correlation with polysomnography [6, 23], it may still need to be more accurate. Fourth, CES-D was used as a surrogate marker for depression, but the patients were not necessarily clinically diagnosed or evaluated in this domain. Fifth, to compare sleep fragmentation between WatchPAT and the SMHSQ data, we chose to use the unified top value of 7 for analysis. Therefore, actual numbers of sleep fragmentation were not used for analysis if the episodes/night were 6 or higher.

In this study, we explored three of the many different aspects of sleep before and after surgery, along with several factors which may influence sleep quality during these periods. Given the importance of qualified sleep to facilitate patient recovery, it is essential to devise ways to measure sleep reliably and objectively in the perioperative setting.

In conclusion, its agreements and a good to fair correlation between the SMHSQ and WatchPAT were observed preoperatively, and both seem reliable for evaluating sleep characteristics before surgery. However, that was not the case for postoperative sleep. Our study indicates that factors that affect cognition and sleep, such as admission type (inpatient vs. outpatient), depression, anesthesia type (general anesthesia vs. other), and sleep apnea, may be related to this discrepancy. Therefore, relying solely on one sleep assessment method is not advisable. Future studies are required both to validate our findings and investigate the etiology of the discrepancy between these two methods.

## Figures and Tables

**Table 1:** Demographic and clinical variables at baseline and intraoperative variables.

Characteristic	*n* = 101
*Demographic variable*	
Male	60 (59.4%)
Age (years)	58.1 ± 11.3
≥65	28 (27.7%)
White	78 (77.2%)
Hispanic	4 (4.0%)
Admission type	
Inpatient	72 (71.3%)
Outpatient	29 (28.7%)

*Clinical variable*	
CES-D	9.4 ± 7.8
Depression (CES − D ≥ 16)	19 (20.9%)
BMI (kg/m^2^)	28.9 ± 6.0
Obese (BMI ≥ 30)	33 (32.7%)
pAHI	15.3 ± 14.2
pAHI ≥ 15	32 (34.0%)

*Intraoperative variable*	
ASA classification	
I	19 (18.8%)
II	65 (64.4%)
III	17 (16.8%)
Surgery time (minutes)	136.2 ± 99.7
Had intraoperative complication	7 (6.9%)
Anesthesia type^*α*^	
General	60 (59.4%)
Other	41 (40.6%)

Data are presented as mean ± standard deviation or *n* (%). CES-D: Center for Epidemiologic Studies Depression scale; BMI: body mass index; pAHI: peripheral arterial tonometry-derived apnea-hypopnea index; ASA: American Society of Anesthesiologists physical status classification. ^*α*^Patients can receive different and multiple types of anesthesia. Others include spinal, epidural, and peripheral nerve blocks except general anesthesia.

**Table 2:** Correlations of sleep fragmentation, sleep latency, and sleep time between WatchPAT and SMHSQ.

	Variable	WatchPAT	SMHSQ	*p* ^∗^	*r*	*p*	Partial *r*	*p*
Preoperative	Sleep fragmentation (times/night)	5.9 ± 1.2 (2-7)	2.7 ± 1.4 (0-7)	<0.001	0.31 [0.10, 0.49]	0.004	0.34 [0.12, 0.53]	0.003
	Sleep latency (min)	20.9 ± 9.6 (5-63)	26.9 ± 23.9 (1-150)	0.014	0.34 [0.14, 0.52]	0.001	0.33 [0.11, 0.52]	0.004
	Night sleep time (hours)	6.0 ± 1.0 (1.3-8.0)	6.9 ± 1.4 (4.0-11.7)	<0.001	0.35 [0.15, 0.52]	<0.001	0.33 [0.11, 0.52]	0.005

Postoperative	Sleep fragmentation (times/night)	6.4 ± 1.3 (1-7)	4.2 ± 2.2 (0-7)	<0.001	0.00 [-0.23, 0.23)	0.969	-0.10 [-0.34, 0.16]	0.464
	Sleep latency (min)	25.0 ± 16.6 (5-73)	28.6 ± 46.2 (0-300)	0.519	-0.16 [-0.38, 0.09]	0.213	-0.03 [-0.30, 0.24]	0.805
	Night sleep time (hours)	6.1 ± 1.5 (1.9-9.3)	5.4 ± 2.2 (0-11)	0.059	0.09 [-0.14, 0.32]	0.431	-0.01 [-0.27, 0.25]	0.925

Data are presented as mean ± standard deviation (range) as well as correlation and 95% confidence interval. *n* = 101. SMHSQ: St. Mary's Hospital Sleep Questionnaire; WatchPAT (Itamar Medical, Caesarea, Israel): portable sleep monitoring device; *r*: Pearson's correlation; Partial *r*: partial correlation. Partial correlation was computed and adjusted for admission type, anesthesia type, depression, obesity, and pAHI. ^∗^*p* value was obtained from paired *t* test to compare the mean values between WatchPAT and SMHSQ.

**Table 3:** Results from the mixed-effects model for sleep fragmentation, sleep latency, and sleep time.

Characteristic	WatchPAT		SMHSQ	
	Beta (95% CI)	*p*	Beta (95% CI)	*p*
*Sleep fragmentation (times)*				
Intercept	6.64 [5.94, 7.33]	<0.001	2.15 [1.13, 3.17]	<0.001
Time: postoperative vs. preoperative	0.50 [0.13, 0.87]	0.007	1.50 [0.96, 2.04]	<0.001
Admission type: inpatient vs. outpatient	-0.79 [-1.34, -0.24]	0.005	0.58 [-0.21, 1.38]	0.15
Anesthesia: general vs. other	-0.60 [-1.12, -0.08]	0.025	0.14 [-0.60, 0.88]	0.7
CES − D ≥ 16 vs. <16	-0.09 [-0.64, 0.45]	0.7	-0.91 [-1.67, -0.14]	0.020
Obesity: BMI ≥ 30 vs. <30	0.11 [-0.42, 0.64]	0.7	0.37 [-0.39, 1.12]	0.3
pAHI: ≥15 vs. <15	0.33 [-0.18, 0.83]	0.2	0.11 [-0.60, 0.83]	0.8

*Sleep latency (min)*				
Intercept	18.99 [11.70, 26.28]	<0.001	24.80 [5.23, 44.36]	0.013
Time: postoperative vs. preoperative	3.86 [0.17, 7.54]	0.040	1.31 [-10.16, 12.78]	0.8
Admission type: inpatient vs. outpatient	1.07 [-4.74, 6.89]	0.7	2.14 [-12.94, 17.22]	0.8
Anesthesia: general vs. other	0.52 [-4.97, 6.01]	0.9	-7.00 [-21.04, 7.05]	0.3
CES − D ≥ 16 vs. <16	5.41 [-0.30, 11.13]	0.063	12.20 [-2.46, 26.86]	0.10
Obesity: BMI ≥ 30 vs. <30	-6.08 [-11.64, -0.52]	0.032	15.15 [0.63, 29.67]	0.041
pAHI: ≥15 vs. <15	4.05 [-1.23, 9.33]	0.13	-7.21 [-20.79, 6.38]	0.3

*Night sleep time (hours)*				
Intercept	5.30 [4.68, 5.93]	<0.001	7.12 [6.14, 8.10]	<0.001
Time: postoperative vs. preoperative	0.03 [-0.30, 0.37]	0.8	-1.51 [-2.07, -0.94]	<0.001
Admission type: inpatient vs. outpatient	0.76 [0.26, 1.25]	0.003	-0.56 [-1.31, 0.19]	0.15
Anesthesia: general vs. other	0.56 [0.09, 1.03]	0.019	0.46 [-0.24, 1.16]	0.2
CES − D ≥ 16 vs. <16	-0.21 [-0.70, 0.27]	0.4	-0.41 [-1.14, 0.32]	0.3
Obesity: BMI ≥ 30 vs. <30	-0.54 [-1.01, -0.06]	0.026	-0.69 [-1.40, 0.02]	0.057
pAHI: ≥15 vs. <15	0.29 [-0.16, 0.74]	0.2	0.70 [0.02, 1.37]	0.042

SMHSQ: St. Mary's Hospital Sleep Questionnaire; BMI: body mass index (kg/m^2^); CI: confidence interval; CES-D: Center for Epidemiologic Studies Depression scale; pAHI: peripheral arterial tonometry-derived apnea-hypopnea index. A mixed-effects model was conducted adjusting for adjusted for admission type, anesthesia type, depression, obesity, and pAHI.

## Data Availability

The data that support the findings of this study are available from the corresponding author upon reasonable request.

## Appendix

St. Mary's Hospital Sleep Questionnaire [4].

## References

[1] Gogenur I., Wildschiotz G., Rosenberg J. (2008). Circadian distribution of sleep phases after major abdominal surgery. *British Journal of Anaesthesia*.

[2] Rosenberg-Adamsen S., Kehlet H., Dodds C., Rosenberg J. (1996). Postoperative sleep disturbances: mechanisms and clinical implications. *British Journal of Anaesthesia*.

[3] Murphy F., Bentley S., Ellis B. W., Dudley H. (1977). Sleep deprivation in patients undergoing operation: a factor in the stress of surgery. *British Medical Journal*.

[4] Ellis B. W., Johns M. W., Lancaster R., Raptopoulos P., Angelopoulos N., Priest R. G. (1981). The St. Mary's Hospital sleep questionnaire: a study of reliability. *Sleep*.

[5] Lewinsohn P. M., Seeley J. R., Roberts R. E., Allen N. B. (1997). Center for epidemiologic studies depression scale (CES-D) as a screening instrument for depression among community-residing older adults. *Psychology and Aging*.

[6] Pang K. P., Gourin C. G., Terris D. J. (2007). A comparison of polysomnography and the WatchPAT in the diagnosis of obstructive sleep apnea. *Otolaryngology and Head and Neck Surgery*.

[7] Zou D., Grote L., Peker Y., Lindblad U., Hedner J. (2006). Validation a portable monitoring device for sleep apnea diagnosis in a population based cohort using synchronized home polysomnography. *Sleep*.

[8] Cronin A. J., Keifer J. C., Davies M. F., King T. S., Bixler E. O. (2001). Postoperative sleep disturbance: influences of opioids and pain in humans. *Sleep*.

[9] Knill R. L., Moote C. A., Skinner M. I., Rose E. A. (1990). Anesthesia with abdominal surgery leads to intense REM sleep during the first postoperative week. *Anesthesiology*.

[10] Dobing S., Frolova N., McAlister F., Ringrose J. (2016). Sleep quality and factors influencing self-reported sleep duration and quality in the general internal medicine inpatient population. *PLoS One*.

[11] Wesselius H. M., van den Ende E. S., Alsma J. (2018). Quality and quantity of sleep and factors associated with sleep disturbance in hospitalized patients. *JAMA Internal Medicine*.

[12] Tsuno N., Besset A., Ritchie K. (2005). Sleep and depression. *The Journal of Clinical Psychiatry*.

[13] Burman D. (2017). Sleep disorders: insomnia. *FP Essent*.

[14] Xiao Q., Gu F., Caporaso N., Matthews C. E. (2016). Relationship between sleep characteristics and measures of body size and composition in a nationally-representative sample. *BMC Obesity*.

[15] Yalamanchali S., Farajian V., Hamilton C., Pott T. R., Samuelson C. G., Friedman M. (2013). Diagnosis of obstructive sleep apnea by peripheral arterial tonometry. *JAMA Otolaryngology. Head & Neck Surgery*.

[16] Closs S. J. (1992). Patients' night-time pain, analgesic provision and sleep after surgery. *International Journal of Nursing Studies*.

[17] Mouch C. A., Baskin A. S., Yearling R., Miller J., Dossett L. A. (2020). Sleep patterns and quality among inpatients recovering from elective surgery: a mixed-method study. *The Journal of Surgical Research*.

[18] Jarad N. A., Sequeiros I. M., Patel P., Bristow K., Sund Z. (2012). Fatigue in cystic fibrosis. *Chronic Respiratory Disease*.

[19] Kuratsune H., Umigai N., Takeno R., Kajimoto Y., Nakano T. (2010). Effect of crocetin from Gardenia Jasminoides Ellis on sleep: a pilot study. *Phytomedicine*.

[20] Ozone M., Yagi T., Itoh H. (2008). Effects of zolpidem on cyclic alternating pattern, an objective marker of sleep instability, in Japanese patients with psychophysiological insomnia: a randomized crossover comparative study with placebo. *Pharmacopsychiatry*.

[21] Davis N., Lee M., Lin A. Y. (2014). Postoperative cognitive function following general versus regional anesthesia: a systematic review. *Journal of Neurosurgical Anesthesiology*.

[22] Ghoneim M. M., O'Hara M. W. (2016). Depression and postoperative complications: an overview. *BMC Surgery*.

[23] Pittman S. D., Ayas N. T., MacDonald M. M., Malhotra A., Fogel R. B., White D. P. (2004). Using a wrist-worn device based on peripheral arterial tonometry to diagnose obstructive sleep apnea: in-laboratory and ambulatory validation. *Sleep*.

